# Impact of closing an emergency department on a neighbouring teaching hospital: the concentrate effect

**DOI:** 10.1186/cc12195

**Published:** 2013-03-19

**Authors:** J Millar, R Wilson, P O'Connor, R McLaughlin

**Affiliations:** 1 Royal Victoria Hospital, Belfast, UK

## Introduction

Closure of an acute hospitals emergency department (ED) has important ramifications for those centres expected to take up the resultant workload. The continued reconfiguration of emergency care is likely to produce an increasing number of these scenarios. Little evidence is available to support planning of such initiatives and thus the implications are difficult to anticipate. This study aims to demonstrate one hospital's experience of the rationalisation of emergency care and its effect on workload.

## Methods

This retrospective study was conducted in a large teaching hospital. Activity data were analysed for a 12-month period following the closure of a neighbouring ED. The results were subsequently compared against the year prior to closure. Attendance, triage data, admission rates and waiting times were compared across the two periods, as were workload data for all grades of physician. The chi-squared test was used to examine differences between groups.

## Results

In the period studied, the gross attendance figure increased by 20,480 (33.72%), whilst the admission rate rose from 22 to 27%. Following closure of the neighbouring ED, the proportion of high-acuity patients attending our institution increased dramatically, with the proportion of category one and two patients (Manchester Triage Scale) increasing by 8.33% (*P *= 0.076) and 18.80% (*P <*0.001), respectively. Likewise, ambulance arrivals increased out of proportion to the total increase in attendances (*P *= 0.016). Admissions from the ED to the ICU increased by 63.04%. Consultants workloads now include 50% more category 1 and 2 patients (*P *= 0.001).

## Conclusion

Reconfiguration of emergency care can have dramatic implications for existing services; these may not always be anticipated. Rationalisation of ED's may result in a concentration of high-acuity patients accompanied by a downturn in the numbers of patients whose presentations are amenable to care delivered in other settings. This abrupt change in case mix requires a re-examination of existing workforces and their seniority.

